# Multifunctional Roles of the Ventral Stream in Language Models: Advanced Segmental Quantification in Post-Stroke Aphasic Patients

**DOI:** 10.3389/fneur.2018.00089

**Published:** 2018-02-27

**Authors:** Jie Zhang, Xuehu Wei, Sangma Xie, Zhen Zhou, Desheng Shang, Renjie Ji, Yamei Yu, Fangping He, Yue Du, Xiangming Ye, Benyan Luo

**Affiliations:** ^1^Department of Rehabilitation Medicine, Zhejiang Provincial People’s Hospital, People’s Hospital of Hangzhou Medical College, Hangzhou, China; ^2^Department of Neurology and Brain Medical Centre, The First Affiliated Hospital, Zhejiang University, Hangzhou, China; ^3^Key Laboratory for NeuroInformation of the Ministry of Education, School of Life Science and Technology, University of Electronic Science and Technology of China, Chengdu, China; ^4^College of Life Information Science and Instrument Engineering, Hangzhou Dianzi University, Hangzhou, China; ^5^Department of Computer Science, Zhejiang University, Hangzhou, China; ^6^Department of Radiology, The First Affiliated Hospital, Zhejiang University, Hangzhou, China; ^7^School of Medicine, Zhejiang University, Hangzhou, China

**Keywords:** aphasia, language model, ventral pathway, stroke, diffusion tensor imaging

## Abstract

In the dual-route language model, the dorsal pathway is known for sound-to-motor mapping, but the role of the ventral stream is controversial. With the goal of enhancing our understanding of language models, this study investigated the diffusion characteristics of candidate tracts in aphasic patients. We evaluated 14 subacute aphasic patients post-stroke and 11 healthy controls with language assessment and diffusion magnetic resonance imaging. Voxel-based lesion-symptom mapping found multiple linguistic associations for the ventral stream, while automated fiber quantification (AFQ) showed, *via* reduced fractional anisotropy (FA) and axial diffusivity with increased radial diffusivity (all corrected *p* < 0.05), that the integrity of both the left dorsal and ventral streams was compromised. The average diffusion metrics of each fascicle provided by AFQ also confirmed that voxels with significant FA-language correlations were located in the ventral tracts, including the left inferior fronto-occipital fascicle (IFOF) (comprehension: *r* = 0.839, *p* = 0.001; repetition: *r* = 0.845, *p* = 0.001; naming: *r* = 0.813, *p* = 0.002; aphasia quotient: *r* = 0.847, *p* = 0.001) and uncinate fascicle (naming: *r* = 0.948, *p* = 0.001). Furthermore, point-wise AFQ revealed that the segment of the left IFOF with the strongest correlations was its narrow stem. The temporal segment of the left inferior longitudinal fascicle was also found to correlate significantly with comprehension (*r* = 0.663, *p* = 0.03) and repetition (*r* = 0.742, *p* = 0.009). This preliminary study suggests that white matter integrity analysis of the ventral stream may have the potential to reveal aphasic severity and guide individualized rehabilitation. The left IFOF, specifically its narrow stem segment, associates with multiple aspects of language, indicating an important role in semantic processing and multimodal linguistic functions.

## Highlights

- Both the dorsal and ventral streams lose integrity in post-stroke aphasia.
- Ventral stream associates with many linguistic aspects, not only comprehension.
- Narrow stem segment of inferior fronto-occipital fascicle is pivotal and multimodal.
- Point-wise automated fiber quantification boosts precise quantification for aphasia.

## Introduction

Language is an advanced cortical function, closely associated with social activity and information transmission, and language deficits lead to severely impaired quality of life. Aphasia affects 21–38% of post-stroke patients, and may become chronic among those who do not receive proper rehabilitation (1, 2). To improve the efficacy of language rehabilitation, it is important to precisely quantify neuroimaging markers that correlate with behavioral changes.

Recent studies have challenged traditional theories of the neural mechanisms of language processing (3–5). The classical model, focusing on cortical language regions, including the Broca and Wernicke areas, cannot explain the clinical problems caused by lesions outside the classical regions (6). Brain sample reexaminations of the first two patients with Broca’s aphasia revealed that damage was not restricted to the cortical surface, but also involved deep subcortical white matter (7, 8). A subsequent large-scale study demonstrated that most cerebral infarction lesions involved white matter, and behavioral disorders caused by damaged white matter, including disconnection syndromes, are not uncommon (9). Moreover, damage to several specific white matter tracts was proved to contribute to severe language impairment (7). Multiple cortical regions contribute to language processing, and each region is connected structurally and functionally to others (3). In the contemporary model, the subcortical white matter tracts transmit information and promote collaboration between components of the distributed language network (3, 4).

Recently, research into subcortical white matter has been dramatically enhanced by the use of diffusion tensor imaging (DTI) (10). Two language-relevant white matter pathways have been identified between frontal and temporal regions: the dorsal and ventral pathways (11, 12). The dorsal pathway consists of the superior longitudinal fascicle (SLF) and arcuate fascicle (AF), and is responsible for sound-to-motor mapping (13, 14). Stroke involving these bundles can lead to dysfunction in speech production and a decreased aphasia quotient (AQ) (15–18). The dorsal route was once considered the dominant pathway in linguistic processing, until higher-level language comprehension was found to be mediated by the ventral stream (19), which consists of the inferior fronto-occipital fascicle (IFOF), uncinate fascicle (UF), and inferior longitudinal fascicle (ILF). The IFOF, also named the extreme capsule fiber system, mediates the interaction between temporal and prefrontal brain regions, and contributes to auditory comprehension (12, 20). Additionally, an important role of the left ventral tracts (UF and ILF) has been found in primary progressive aphasia patients with specific semantic deficits (21).

The ventral stream is still deserving of further investigation. Previous studies have explored the main course of the IFOF, but its components and cortical terminations remain unclear (22, 23). Apart from the complicated architecture of the IFOF, evidence for the role of the ventral pathway in language processing has been inconsistent, and the roles of the UF and ILF are controversial (24–26). More detailed quantitative evidence from stroke survivors with linguistic deficits would contribute to enriching the contemporary language model of dual-route white matter streams. Previous DTI studies on post-stroke aphasia have mainly been conducted among chronic patients, and there is less data on patients in the acute or subacute phases (16, 17, 27–30). However, baseline information is particularly valuable for identifying early predictors and comparing changes in recovery. Additionally, most of the previously mentioned studies evaluated global language ability, or a subtest, and thus cannot reflect comprehension ability. Moreover, previous work has focused on only a few tracts, due to the time-consuming nature of manual tractography (15, 17, 30, 31), and diffusion characteristic variations along the trajectory of each fascicle have not been explored in subacute aphasic patients after stroke.

In this study, we scanned aphasic patients during the subacute post-stroke period and assessed their behavioral performance for various aspects of language. As a preliminary investigation, tract-based spatial statistics (TBSS) analyses were performed to locate regions of altered white matter and language-related tracts. In addition, a more precise and convenient tool, automated fiber quantification (AFQ), was adopted to extract mean diffusion values by resampling 100 equidistant nodes along bilateral major fascicles. We hypothesized that different language subcomponents would be related to candidate predictive tracts and critical segments in the dual streams, and that the ventral stream contributes to multimodal processing.

## Materials and Methods

### Participants

We consecutively recruited 14 post-stroke patients with clinically suspected language dysfunction from the First Affiliated Hospital of Zhejiang University, Hangzhou, China between May 2016 and March 2017. Ethical approval was obtained from the Local Research Ethics Committee of the First Affiliated Hospital of Zhejiang University. All patients suffered from first-onset cerebral infarction, and most were enrolled with subacute stroke. The participant inclusion criteria were as follows: (1) diagnosed with left hemisphere infarction confirmed by clinical and magnetic resonance imaging (MRI) evidence; (2) within 1 month of initial stroke episode; (3) right-handed, assessed by the Standard Chinese Handedness Scale; (4) aged >25 years old with an educational level ≥6 years; (5) native Chinese-speaking, and diagnosed with aphasia by the Aphasia Battery of Chinese (ABC) (32). Exclusion criteria included: pre-existing neurologic or psychiatric conditions, global cognitive impairment, and MRI contraindications. Additionally, 11 age-, sex- and education- matched healthy control subjects were also enrolled.

All procedures performed in this study involving human participants were in accordance with the ethical standards of the Local Research Ethics Committee of the First Affiliated Hospital of Zhejiang University (reference number: 2016-314), and all subjects gave written informed consent in accordance with the Declaration of Helsinki.

### Behavioral Data

Neuropsychological assessment, including the ABC language scale and the Mini-Mental State Examination (33), were performed by the same professional examiner within three weeks of aphasia-onset. The ABC language scale consists of multiple subtests, including spontaneous speech, repetition, comprehension, naming, reading, and writing. It was modified from the Western Aphasia Battery (34) and was adapted to Chinese language features. The AQ, reflecting the general performance of oral language, was calculated using scores for spontaneous speech, repetition, and comprehension. Patients were classified into different aphasia types according to the standard procedure (32).

### MRI Protocol

All subjects underwent MRI scans on a 3T Signa HDxt 2.0 GE (General Electric) scanner within 4 weeks of stroke. The imaging protocol included a high-resolution structural T1-weighted volume of the whole brain, acquired using the three-dimensional brain volume imaging sequence (TR = 7.8 ms; TE = 3.0 ms; field of view = 256 mm × 256 mm; matrix size = 256 × 256; flip angle = 7°; 192 slices with no gap; slice thickness = 1 mm). Additionally, diffusion tensor data were sampled using a spin-echo, single-shot, echo-planar imaging sequence (TR = 13,000 ms; TE = 92.0 ms; field of view = 256 mm × 256 mm; matrix size = 128 × 128; flip angle = 7°; 50 slices with no gap; slice thickness = 3 mm; 32 non-collinear diffusion sensitization directions at *b* = 1,000 s/mm^2^ and 3 b0 images).

### Imaging Data Pre-Processing

For structural 3D T1 images, we used the FMRIB Software Library (FSL version 5.0.9,^1^) (35) to conduct rotations to the standard direction. We deleted non-brain tissue from the whole head using the brain extraction tool (BET) (36).

Raw diffusion data were corrected for eddy current distortions and subject head motion by firstly using FMRIB’s diffusion toolbox, and then deleting non-brain tissue with the BET. Whole-brain images of diffusion metrics, including fractional anisotropy (FA), S0 (raw T2 signal with no diffusion weighting), as well as first, second, and third eigenvalues (L1, L2, and L3), were obtained *via* DTIFIT, which was used to calculate the diffusion tensor model at each voxel. Finally, other three common metrics including axial diffusivity (AD), radial diffusivity (RD), and mean diffusivity (MD) (10) were calculated with appropriate formulas: AD = L1, RD = (L2 + L3)/2, MD = (L1 + L2 + L3)/3. At the microstructural level, FA measures the degree of structural integrity and myelination of white matter (37, 38), while AD reduction reflects axonal injury and RD is selectively sensitive to myelin damage (39).

### Analytical Strategy

**Figure 1:**
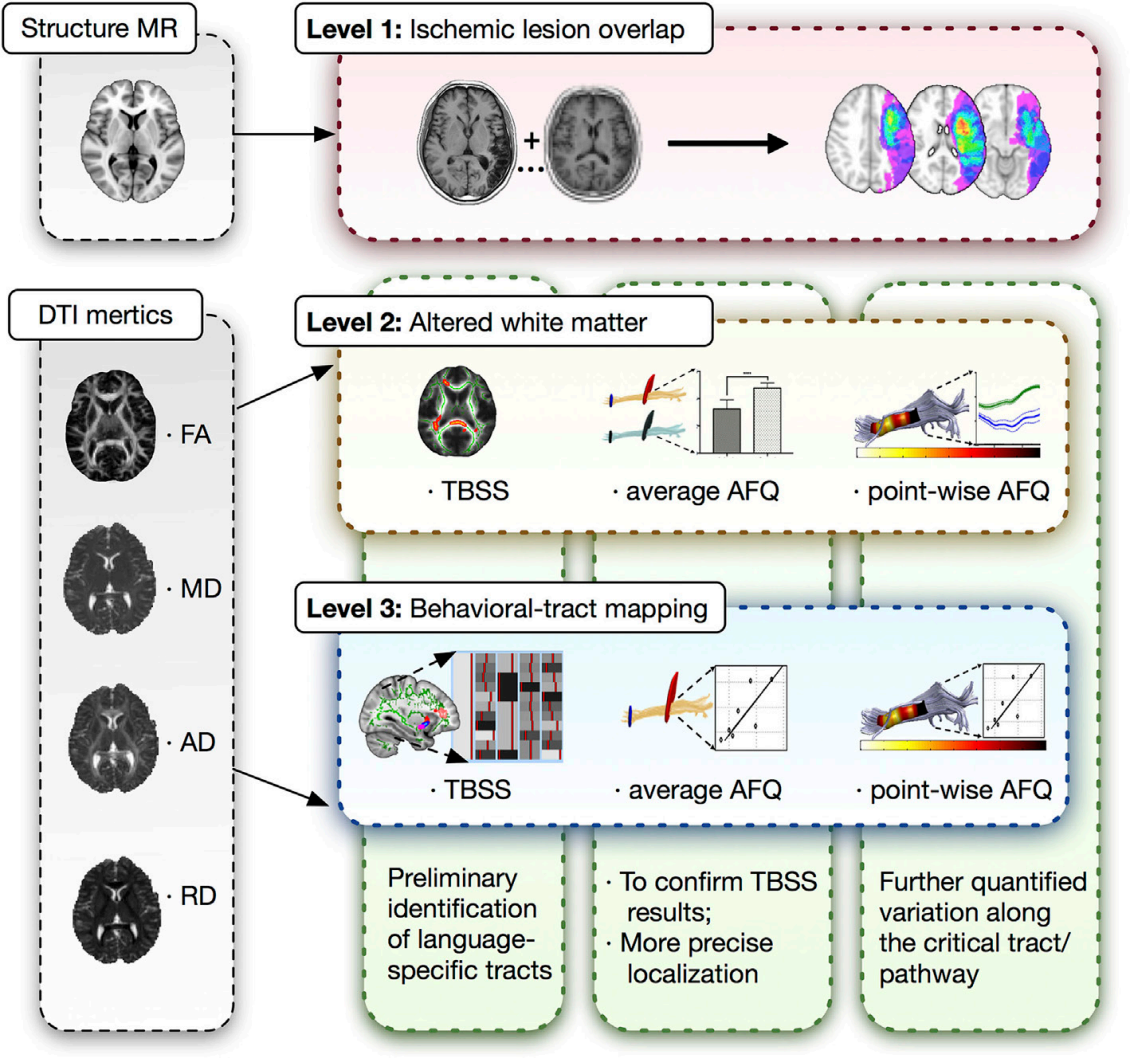
Three-level strategy for neuroimaging analysis. DTI, diffusion tensor imaging; MR, magnetic resonance; AFQ, automated fiber quantification; TBSS, tract-based spatial statistics.

A three-level strategy was designed for this neuroimaging analysis in patients with post-stroke aphasia, which was summarized in Figure 1.

### Lesion Overlap Analysis

The characteristics of ischemic lesions including their location and size were provided by T1-weighted images. The individual lesion regions were delineated manually as binary masks by an experienced neurologist (Jie Zhang) and a radiologist (Desheng Shang), and then normalized into the Montreal Neurological Institute space using FSL. These normalized binary lesion mask were summed to generate a lesion overlay maps. Lesion size was obtained by calculating the voxel number of individual lesion masks.

### Tract-Based Spatial Statistics

Voxelwise statistical analysis of the diffusion data including FA and non-FA (AD, RD, and MD) images was carried out using TBSS (40). All subjects’ FA data were normalized to the FMRIB58 FA template, aligned into a common space using both linear and non-linear registration. Next, the mean FA image was created and thinned to create a mean FA skeleton which represents the centers of all tracts common to the group. Each subject’s aligned FA and non-FA data were then projected onto this skeleton and the resulting data fed into voxel-wise cross-subject statistics. Comparisons of diffusion metrics between patients and controls were tested by using a two-sample *t*-test and were adjusted for additional covariates including age and sex. Correlations between diffusion metrics and behavioral scales were tested based on the general linear model, controlling above additional covariates. The number of permutations was set at 10,000. The resulting statistical maps were corrected for multiple comparisons, i.e., the family-wise error correction using the threshold-free cluster enhancement option. The threshold for statistical significance was *p* < 0.05. The Johns Hopkins University White Matter Tractography Atlas was used for identification of tracts (41).

### Automated Fiber Quantification

We performed AFQ (version 1.2; Stanford University, California, USA;^2^) to enable further quantitative analysis along the major tracts. Automated calculation was carried out using MATLAB (version R2014a; the MathWorks, Inc., Natick, Massachusetts, USA). We used dtiMakeDt6^3^ to convert S0 images into dt6.mat files. Six steps were required to obtain the AFQ results (42). Firstly, whole-brain tractography fiber using a deterministic streamlines tracking algorithm (43) was initiated from each white matter voxel with FA >0.3. Two tracking termination criteria were set: (1) FA <0.2 and (2) angle between two consecutive tractography steps >45°. Secondly, the trajectory of fascicles was defined by two waypoint regions of interest (ROIs) referring to previous studies (44). The third step was fiber tract refinement, which was accomplished by comparing each candidate fiber to fiber tract probability maps (41). Next, fiber tract cleaning was performed by removing fibers that were more than four SDs above the mean fiber length or that deviated more than five SDs from the core of the fiber tract. The fiber group was clipped to the central portion that spans between the two defining ROIs. Finally, 100 equidistant nodes were resampled along the central portion of the tract in each fiber group, and the mean location of each node in the fiber group core was calculated. The final quantified fiber group was labeled the “Tract Diffusion Profile,” containing a vector of 100 values representing the diffusion properties (42). Based on preliminary TBSS analyses, we chose the left major fascicles including SLF, AF, IFOF, UF, and ILF as target tracts for AFQ.

### Statistical Analysis

Independent two-sample *t*-tests were performed to compare the difference between aphasic patients and healthy controls for mean diffusion metrics in major white matter fascicles. The language scores of each subtest were all transformed into *Z*-scores that fitted normal distribution. The relationships between tract-specific diffusion metrics and specific language *Z*-scores were assessed using Pearson’s correlation coefficients. The corrected significant threshold for mean diffusion metrics and point-wise univariate statistics of each fascicle was *p* < 0.05 with Bonferroni correction. Age, gender, and lesion size were listed as nuisance covariates in correlation analyses. *Post hoc* power calculation was performed by G*power software (Version 3.1.9.2 for Mac), and (1 − β) > 0.8 means enough power for the statistical test.

## Results

### Demographic Data and Language Characteristics

Fourteen patients with post-stroke aphasia were enrolled based on the ABC language scale and MRI scans, including the DTI sequence. All were right-handed and suffered from cerebral infarction located in the left hemisphere. Most of them were at the subacute phase of first-onset stroke. Language function was assessed 10 ± 2 days after onset, and MRI scans were completed after 14 ± 2 days. Two patients were removed because of obvious head motion, and one sample with damaged data storage was excluded. Eleven age- and sex-matched healthy control subjects were also assessed. The demographic data of the two groups are shown in Table 1. There was no statistically significant between-group difference for age, sex, or education level between patients and healthy volunteers (*p* > 0.05).

**Table 1:** Demographics and language scores for aphasic patients at baseline.

	Aphasic patients	Healthy controls
**Demographics**		
Age/years, median (range)	62 (28–67)	50 (25–80)
Education/years, median (range)	9 (6–16)	11 (6–18)
Sex, male (%)	63.6	45.5
Handedness, right (%)	100	100
**Interval after onset/days, median (range)**		
Time to language test	11 (1–21)	–
Time to scan/days	13 (5–27)	–
**Language performance^a^, median (range)**		
Comprehension	25.22 (13.91–83.48)	–
Repetition	6 (0–89.00)	–
Naming	0 (0–87.10)	–
Reading	6.67 (0.83–80)	–
Writing	0 (0–55.24)	–
Aphasia quotient	30.59 (18.48–73.58)	–
**Aphasia type (%)**		
Global	63.6	–
Transcortical motor	18.2	–
Broca	9.1	–
Anomic	9.1	–

*^a^All the language subtest scores were converted into a hundred-mark system*.

The ABC language results showed that the average performance of each subtest was lower than 40%, indicating obviously damaged language function. Most patients (63.6%) had global aphasia with multiple dysfunction covering oral and written aspects (Table 1).

### Lesion Distribution Analysis

**Figure 2:**
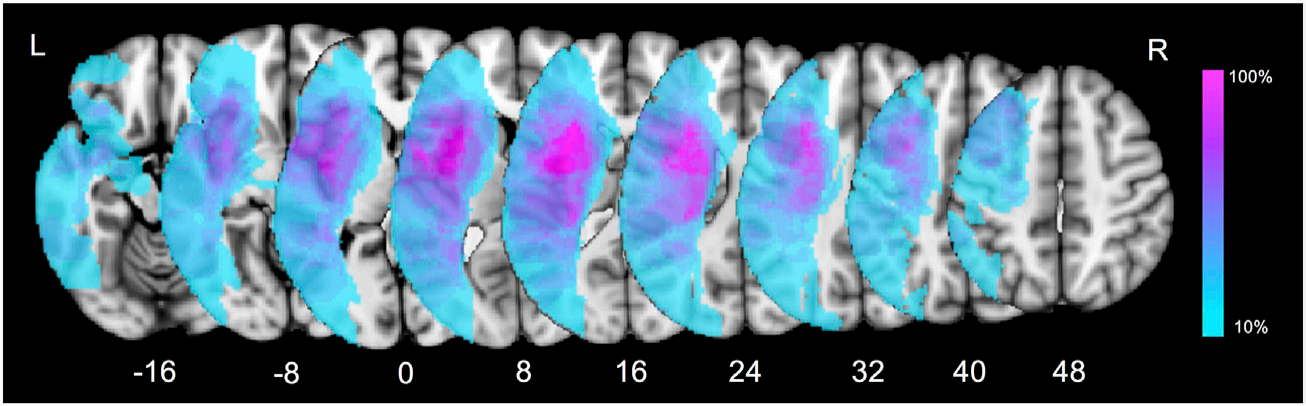
Lesion overlay map for all the aphasic patients. Color bar indicates percentage of patients with lesions in a particular voxel (0–100%).

Firstly, a lesion overlay map was performed to present the characteristics of ischemic lesion distribution in our patient group (Figure 2). The left insula and opercular cortex were the most commonly damaged cortical regions. As for the subcortical level, the highest overlay was located at the putamen, internal capsule, the external/extreme capsule, claustrum, and the white matter around the temporoparietal junction. The overlap percentage result indicated that subcortical structures were considerably susceptible in post-stroke patients with language impairment.

### Identification of Ventral Tracts Correlated with Language

**Figure 3:**
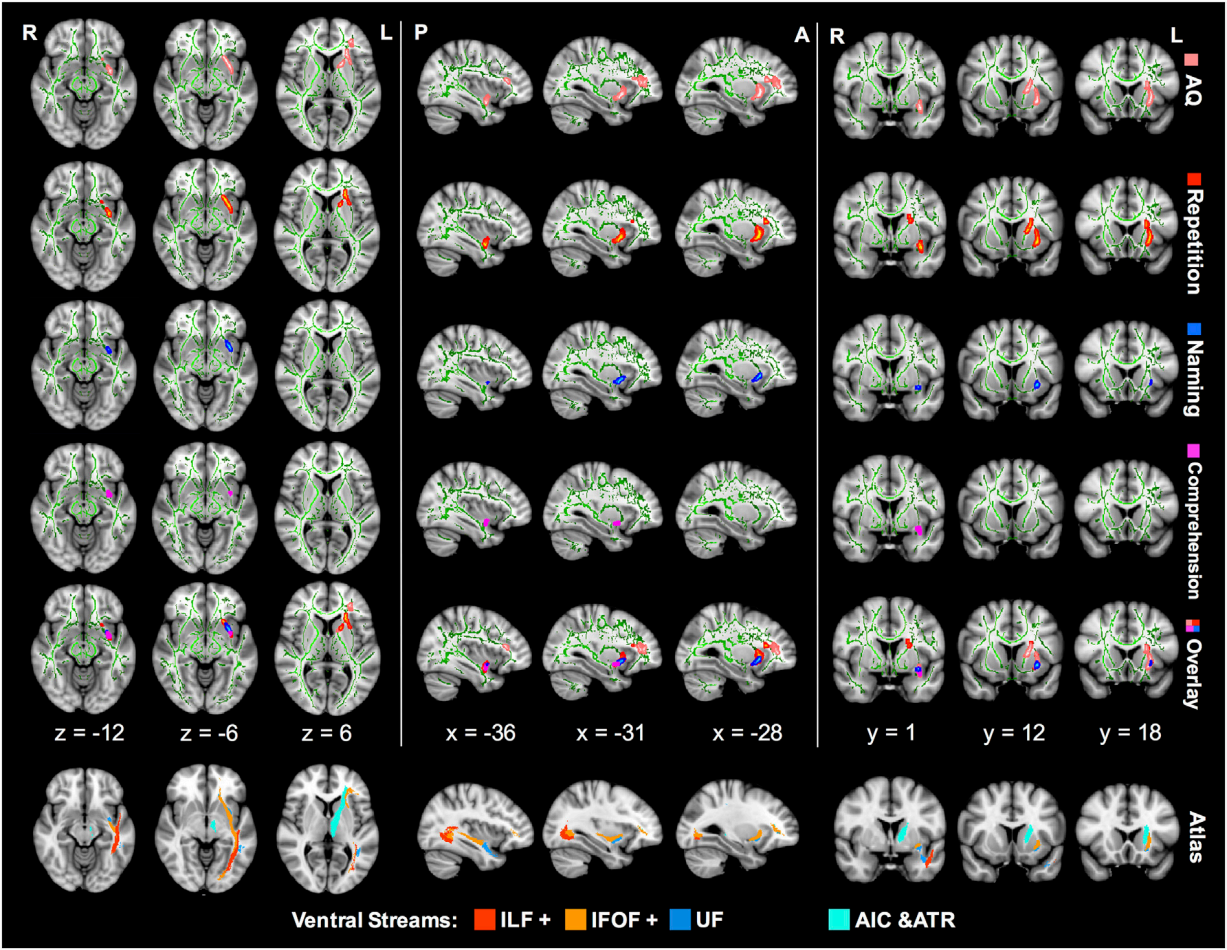
TBSS correlation analysis between the FA value and language subcomponents in aphasic patients. The green lines in every image represent a mean white matter skeleton. The regions in pink stand for AQ-related tracts with significant positive correlation (*p* < 0.05; TFCE corrected). Similarly, regions of significant positive correlation with repetition are in red, naming in blue, and comprehension in magenta. All the correlations were controlled by additional covariates including age, sex, and lesion size. TBSS, tract-based spatial statistics; IFOF, inferior fronto-occipital fascicle; ILF, inferior longitudinal fascicle; UF, uncinate fascicle; AIC, anterior internal capsule; ATR, anterior thalamic radiation; AQ, aphasia quotient; TFCE, threshold-free cluster enhancement; FA, fractional anisotropy.

Tract-based spatial statistics correlation analyses showed preliminary lesion-language mapping, locating language-specific tracts in the ventral pathway (Figure 3). The left IFOF was the most remarkable tract where the FA value showed significantly positive correlations with multiple language subtests.

*Z*-score of repetition correlated positively with FA of the left IFOF in the ventral external capsule, isthmus of the UF, and anterior internal capsule (AIC) (corrected *p* < 0.05). The AQ-related regions in the FA map were similar to the repetition distribution, and the corresponding IFOF extended further into the frontal lobe. In contrast, comprehension and naming scores were associated with FA in smaller regions excluding the AIC. In the overlap mode of Figure 3, the region of significant FA-comprehension correlation was more posterior and ventral in spatial distribution than repetition, and the naming-related region was located in-between.

However, there were no statistically significant correlations between language subtests and other diffusion metrics, including AD, RD, and MD (corrected *p* > 0.05).

### Group Difference of from White Matter Average and Point-Wise Levels

Between-group difference of white matter alterations was confirmed by mean diffusion measures with AFQ (Table 2). Apart from ventral tracts, dorsal tracts were also investigated for comparison. Multiple diffusion metrics of the left IFOF in aphasic patients changed significantly, including reduced FA (0.32 ± 0.07 vs 0.48 ± 0.03), reduced AD (1.19 ± 0.13 vs 1.35 ± 0.04), and increased RD (0.72 ± 0.08 vs 0.60 ± 0.05) compared with healthy controls (all corrected *p* < 0.05). For the left ILF, FA, and RD changed significantly with no obvious alterations in AD or MD; in the left AF and UF, only reductions in FA were recorded among the four diffusion metrics (all corrected *p* < 0.05).

**Table 2:** Group differences of regional mean diffusion tensor indices in the tracts of interest.

Tracts	FA, mean (SD)		AD, mean (SD)		RD, mean (SD)	
	Patient	Control	Patient	Control	Patient	Control
SLF	0.29 (0.05)^a^	0.37 (0.04)	1.05 (0.15)	1.09 (0.08)	0.68 (0.11)	0.60 (0.04)
AF	0.33 (0.06)^a^	0.47 (0.03)	1.08 (0.16)	1.21 (0.05)	0.64 (0.10)	0.56 (0.04)
IFOF	0.32 (0.07)^a^	0.48 (0.03)	1.19 (0.13)^a^	1.35 (0.04)	0.72 (0.08)^a^	0.60 (0.05)
UF	0.34 (0.07)^a^	0.43 (0.03)	1.16 (0.13)	1.27 (0.06)	0.67 (0.03)	0.62 (0.04)
ILF	0.36 (0.03)^a^	0.42 (0.02)	1.27 (0.07)	1.29 (0.05)	0.71 (0.05)^a^	0.65 (0.03)

*Values are expressed as mean (SD); AD and RD are measured in square millimeter per second × 10^−3^*.

*^a^Significantly impaired relative to healthy controls at P < 0.05 after Bonferroni correction*.

*FA, fractional anisotropy; AD, axial diffusivity; RD, radial diffusivity; SLF, superior longitudinal fascicle; AF, arcuate fascicle; IFOF, inferior fronto-occipital fascicle; UF, uncinate fascicle; ILF, inferior longitudinal fascicle*.

**Figure 4:**
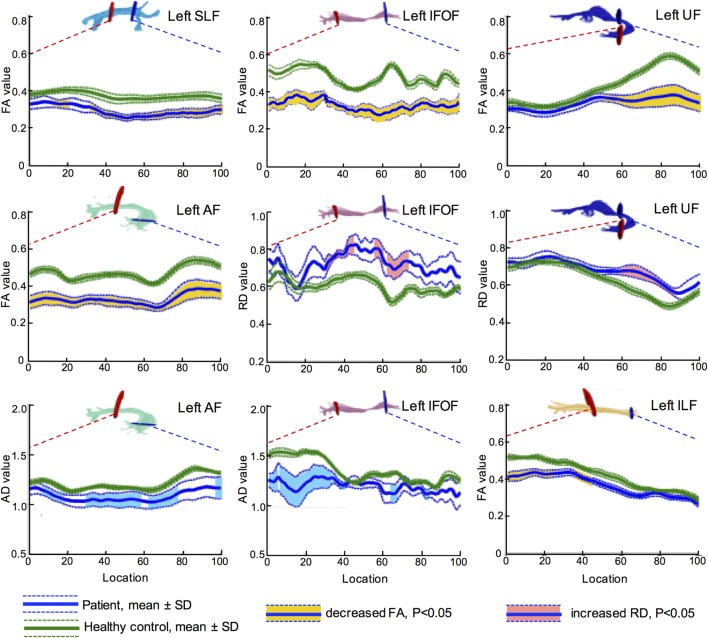
Point-wise comparisons for diffusion measurement along the tract trajectory between patients and controls. The horizontal scale represents 100 equidistant nodes along the central portion of the tract, defined by the red starting point and blue point. The blue solid line refers to the mean value of patient group, while the green solid line refers the mean value of control group. The dash lines stand for their SD. The vertical scale refers to diffusion metrics, and the segments with significant difference are marked in different colors, yellow for FA, light blue for AD, and pink for RD (all corrected *p* < 0.05). Values of AD and RD are measured in square millimeter per second × 10^−3^. FA, fractional anisotropy; RD, radial diffusivity; AD, axial diffusivity; IFOF, inferior fronto-occipital fascicle; ILF, inferior longitudinal fascicle; UF, uncinate fascicle; SLF, superior longitudinal fascicle; AF, arcuate fascicle.

Further, more accurate point-wise comparisons were acquired to investigate the variation along the tract trajectory. As seen in Figure 4, FA of the left IFOF reduced across the entire span (corrected *p* < 0.05). AD in the left IFOF was mainly reduced in the occipital and posterior temporal sections (0–43th point), whilst RD increased in the middle segment (30–46th and 60th–73rd point). As to the left UF, its insular segment of showed remarkable FA and RD alterations.

In summary, decreased FA and increased RD indicated that left major fascicles had lost myelin sheath integrity. Furthermore, axons in the left hemisphere suffered severe damage after stroke, as both AD and FA reduced. The brain edema was not influential in the subacute post-stroke group since MD did not change significantly in both hemispheres (all corrected *p* > 0.05).

### AFQ Evidence of Correlations between Diffusion Metrics and Language Performance

**Figure 5:**
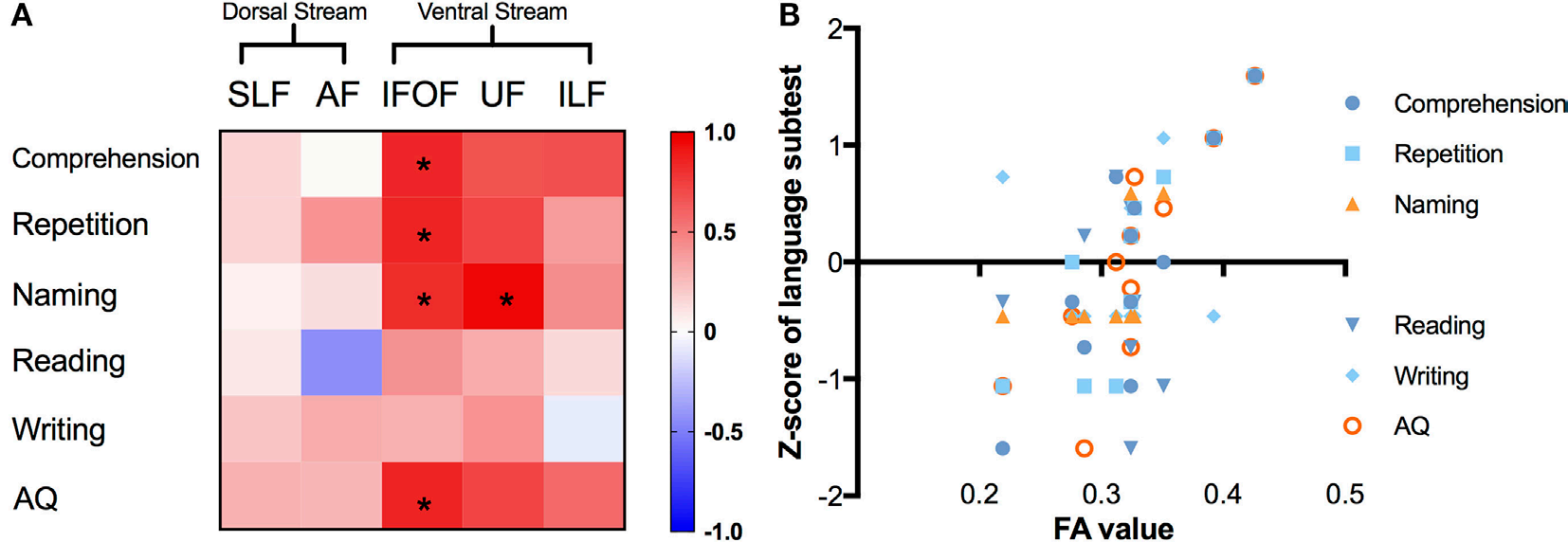
Correlations between different language subcomponents and mean FA value of the left major fascicles. **(A)** Correlation matrix with a color scale, squares in red mean positive correlations while blue ones mean negative correlations, asterisks denote significant correlations (all corrected *p* < 0.05). **(B)** Correlation scatter plots for the left IFOF, presenting the relationships between FA values (*x*-axis) and *Z*-scores of language subtests on the *y*-axis. FA, fractional anisotropy; IFOF, inferior fronto-occipital fascicle; ILF, inferior longitudinal fascicle; UF, uncinate fascicle; SLF, superior longitudinal fascicle; AF, arcuate fascicle; AQ, aphasia quotient.

We summarized the correlations with the average metric of the left major fascicles by AFQ in Figure 5. In the matrix (Figure 5A), correlations of corrected significance were confirmed to be located in the ventral tracts, including the left IFOF and UF (all corrected *p* < 0.05). The mean FA of the left IFOF correlated positively with four different language scores (*r*_comprehension_ = 0.839, *p* = 0.001; *r*_repetition_ = 0.845, *p* = 0.001; *r*_naming_ = 0.813, *p* = 0.002; *r*_AQ_ = 0.847, *p* = 0.001), as seen in Figure 5B. Moreover, there was a strong positive correlation between the mean FA in the left UF and the *Z*-score of the naming subtest (*r* = 0.948, *p* = 0.001).

**Figure 6:**
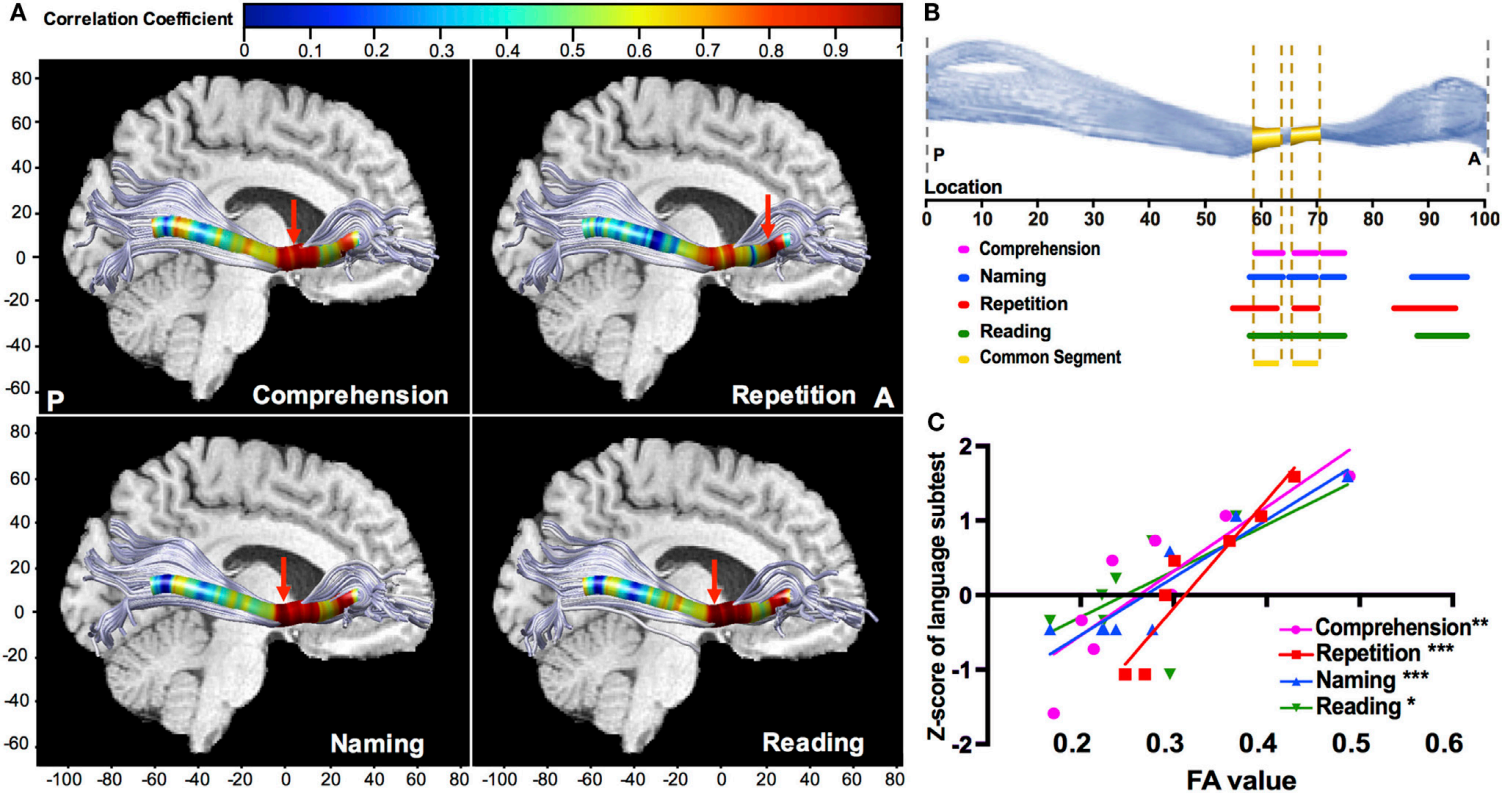
Point-wise correlations along the trajectory of the left IFOF between FA value and language subcomponents. **(A)** Colors correspond to the magnitude of correlations along the 100 equidistant points, and red arrows points out the location of the maximal correlation; **(B)** the sections of significant correlations along the 100 equidistant points of the left IFOF (all corrected *p* < 0.05). For naming (blue), repetition (red), and reading (green), the significant sections are the narrow stem and frontal radiation; the significant sections for comprehension are within the narrow stem; **(C)** scatter plots with the regression curves for the point of maximal correlation, presenting the linear relationships between FA value (*x*-axis) and *Z*-scores of language subtests on the *y*-axis (*p < 0.05; **p < 0.01; ***p < 0.001). FA, fractional anisotropy; IFOF, inferior fronto-occipital fascicle.

Point-wise correlations along the trajectory provided more precise localization in neuroanatomy. The left IFOF provided multiple positive correlations (Figure 6A), and the significant segments with FA were located in its narrow stem (55–75th point) and frontal radiation (84–97th point) (Figure 6B). The narrow stem was the crucial common segment shared by all the significant correlations (Figure 6B). The strongest correlations (Figure 6A, red arrows) with different language subtests of comprehension, naming, and reading were all located in the narrow stem, between the Broca’s and Wernicke’s areas, whilst the strongest correlation with repetition was in the frontal radiation (Figure 6C; *r*_comprehension_ = 0.863, *p* = 0.006; *r*_repetition_ = 0.951, *p* < 0.001; *r*_naming_ = 0.927, *p* < 0.001; *r*_reading_ = 0.727, *p* = 0.04). Moreover, the left ILF showed significant positive correlations with comprehension and repetition in the temporal segment (*r*_comprehension_ = 0.663, *p* = 0.03; *r*_repetition_ = 0.742, *p* = 0.009). Point-wise correlations for other fascicles were also explored, as seen in Figures S1 and S2 in Supplementary Material.

## Discussion

The present study used a combined DTI approach of TBSS and AFQ to investigate the relationship between target fascicles of dual streams and multiple language subcomponents. These two methods could supplement and verify each other to draw more convincing conclusions. Based on comprehensive diffusion indices, TBSS played a role in preliminarily mapping specific language function to the ventral tracts including the left IFOF and UF. Next, group comparison by AFQ was taken as a prior step to ascertain microstructural features of altered white matter and figure out the segment of significant difference. Demyelination and axonal injury were indicated in these white matter tracts, given that FA and AD were reduced combined with increased RD (37, 39). On the other side, there was no evidence for cerebral edema on account of no significant MD changes, excluding its influence on diffusion tensor (45). We further conducted correlation analysis using AFQ, showing that significant correlations along the left IFOF were mainly located in the narrow stem and frontal radiation. Although AFQ have been utilized in previous research (46), our DTI study is the first to use it in subacute post-stroke patients with language deficits. The narrow stem of the left IFOF had the strongest correlation with comprehension, naming, and reading, whereas the frontal radiation presented the maximal repetition-related correlation. Abundant positive results indicated the pivotal role of the left IFOF in multiple tasks of language subcomponents. In addition, AFQ correlation analysis revealed that the left ILF might also contribute to comprehension, whilst the left UF may be related to the process of naming.

Previous language processing theories and research support our main finding, that the left IFOF is involved in multimodal semantic processing (12, 47). The IFOF is the longest associative bundle in the human brain and adjoins distributed brain regions (48). As its name suggests, IFOF connects the frontal lobe, temporobasal areas, and the superior parietal lobe to the frontal lobe, passing through the temporal lobe and insula (22). The anterior part of the left IFOF is near the Broca’s area, including the pars triangularis and opercularis, and its middle temporal segment is located on the roof of the temporal horn (23). It is adjacent to two classical language regions, suggesting the crucial role of the IFOF in the contemporary language model. Additionally, the IFOF is characterized by multiple components. The multifunction feature of the left IFOF for language processing, revealed by stimulation studies, is consistent with its various terminations, including in prefrontal, parietal, and temporal-basal areas (47). Recently, anatomists dissected the IFOF into two components, including the superficial dorsal and deep ventral subcomponents (22). The inferior frontal gyrus and posterior part of the superior temporal gyrus are connected by the superficial layer, indicating the verbal executive aspects of semantic processing. As per AFQ results, the frontal section of the left IFOF showed a relationship with repetition, implying verbal semantics of the superficial component. Meanwhile, the deep and ventral layers are associated with the fusiform area, known as visual word form area (23). Interestingly, this may interpret the positive correlation between reading level and FA in some loci of the left IFOF. Correlations with naming and reading performance were also consistent with brain electrostimulation studies, indicating that the deep layer of the left IFOF is involved in visual recognition and conceptual perception (49).

The IFOF narrows at the frontotemporal junction as it passes through the external capsule (49), connecting the posterior and superior temporal areas and inferior frontal cortex/dorsolateral prefrontal cortex, the two essential epicenters in the dominant hemisphere (50). This supports our results; both the TBSS and AFQ analyses correlated comprehension level with fiber integrity of the left IFOF. Furthermore, from the perspective of this study, the strongest correlation in the narrow stem of the left IFOF confirmed the concordance between the functionally responsible segment and the anatomic locations where two essential epicenters of the semantic network conjoin.

Both auditory information processed at the temporal and parietal cortices and visual information processed at the level of the occipital and temporal-basal cortices, are transmitted to the prefrontal areas in the amodal form (47). In a sense, the left IFOF unifies the multimodal stimuli from the Geschwind’s territory, and is potentially the region responsible for processing both verbal and non-verbal semantics. Playing a pivotal role in the ventral language pathway, the measurement of the left IFOF is expected to facilitate invidualized rehabilitation through precise post-stroke evaluation. For instance, aphasic patients whose left IFOF is severely damaged may require intensive speech therapy and combined interventional treatment.

In parallel with the direct pathway of the IFOF, the UF and the anterior part of the ILF constitute the indirect pathway of the ventral stream (47, 51). Our results also provide evidence for the indirect pathway. The positive correlation with the average FA value demonstrates that the UF might be involved in information processing during naming tasks. Anatomically, the UF runs in front of the IFOF, linking the ventrally located frontal operculum (FOP) with the anterior temporal cortex (52). Whether the UF plays a role in the language model is debated. It was traditionally suggested the UF supported language processing in overall stages, but it was difficult to clarify its specific responsibility (24, 53, 54). The naming function of the UF, especially its role in the retrieval of word form for proper names, is supported by a neurosurgical study in which the resection of the UF led to difficulty in the task of naming famous faces (55). However, another neuroimaging study provided evidence that the UF was involved in the syntactic processes (56), concordant with the syntactic responsibility of its frontal termination, the FOP. There was also doubt about the language function of the UF, given that intraoperative stimulation did not cause naming or semantic disorders (51, 57). As for the ILF, the structural connection with the anterior and inferior posterior temporal lobes indicates its relationship with semantic processing (58, 59). Our finding is consistent with the evidence that therapy-related ILF plasticity was related to semantic improvements (46). Besides, another DTI study supported the semantic involvement of the ILF on account of its predictive ability for word-picture matching performance (29). In summary, the current study proposes positive evidence for the UF and ILF from aphasic stroke survivors, which needs further confirmation by large-scale and multimodal research in the future.

In contrast, both the left SLF and AF in the dorsal stream showed no significant correlations from evidence of both the TBSS and AFQ, though fiber integrity in the axons of the dorsal bundles was clearly damaged. The phenomena are consistent with previous DTI studies. However, these studies also investigated correlations with the remaining volume or lesion load of dorsal bundles, reporting relationships with global severity, and speech production (15–17). Thus, morphologic indices would be adopted in future studies, and follow-up assessment with subtler scales at the appropriate time is required to reflect oral performance.

There were limitations to the present research. First, the sample of the current aphasic cohort was small. The results are preliminary and need to be proven in a larger representative cohort in the future. Secondly, the types and severity of clinical aphasia were not consistent. This is beneficial for correlational studies but introduces confounding factors. Thirdly, a follow-up study is needed to facilitate further understanding of the theory of language rehabilitation and quantification of the predictive model.

## Conclusion

Both the myelin and axons of white matter tracts in the left dorsal and ventral streams were impaired in aphasic patients after left hemispheric stroke. The reduced white matter integrity of the ventral stream, especially the left IFOF, reflects aphasic severity in multiple language aspects, including comprehension, naming, repetition, and reading. This preliminary study sheds light on the pivotal role of the ventral stream in the contemporary language model and individualized post-stroke rehabilitation based on precise evaluation.

## Ethics Statement

All procedures performed in this study involving human participants were in accordance with the ethical standards of the Local Research Ethics Committee of the First Affiliated Hospital of Zhejiang University (reference number: 2016-314), and all subjects gave written informed consent in accordance with the 1964 Helsinki declaration and its later amendments.

## Author Contributions

JZ designed the study, collected the imaging data, and drafted the manuscript; XW was responsible for analyzing the data, drawing figures, and drafting the manuscript; SX and ZZ analyzed and interpreted the data. RJ and YY were responsible for behavioral assessment and data interpretation. DS set the scanning protocols and also involved in analyzing the data. FH and YD designed the study and supervised the whole procedures. XY and BL were responsible for editing and revision. All the authors contributed to editing of the manuscript.

## Conflict of Interest Statement

The authors declare that the research was conducted in the absence of any commercial or financial relationships that could be construed as a potential conflict of interest.

## Footnotes

^1^www.fmrib.ox.ac.uk/fsl.

^2^https://github.com/jyeatman/AFQ.

^3^https://github.com/vistalab/vistasoft/tree/master/mrDiffusion.

## Abbreviations

DTI, diffusion tensor imaging; AFQ, automated fiber quantification; TBSS, tract-based spatial statistics; FA, fractional anisotropy; MD, mean diffusivity; RD, radial diffusivity; AD, axial diffusivity; IFOF, inferior fronto-occipital fascicle; ILF, inferior longitudinal fascicle; UF, uncinate fascicle; ECFS, extreme capsule fiber system; SLF, superior longitudinal fascicle; AF, arcuate fascicle; AQ, aphasia quotient; ABC, Aphasia Battery of Chinese.
