# Supplementary material for: Multifunctional Roles of the Ventral Stream in Language Models: Advanced Segmental Quantification in Post-Stroke Aphasic Patients
Source: Front Neurol. 2018 Feb 27;9:89. doi: 10.3389/fneur.2018.00089 (PMC5835331; doi:10.3389/fneur.2018.00089)
Supplement: Supplementary file 1 [file Data_Sheet_1.PDF]

Supplementary Material

Multifunctional roles of the ventral stream in language models: advanced segmental quantification in post-stroke aphasic patients

Jie Zhang, Xuehu Wei, Sangma Xie, Zhen Zhou, Desheng Shang, Renjie Ji, Yamei Yu, Fangping He, Yue Du, Xiangming Ye, Benyan Luo*

***Correspondence:** Benyan Luo, E-mail: luobenyan@zju.edu.cn; Tel: +86-13967166677; Fax: 057187235101; Xiangming Ye, E-mail: yexmdr@hotmail.com; Tel: +86-13003668420

Supplementary Figures

Supplementary Figure 1 Point-wise correlations along the trajectory of the left ILF between FA value and language subcomponents.

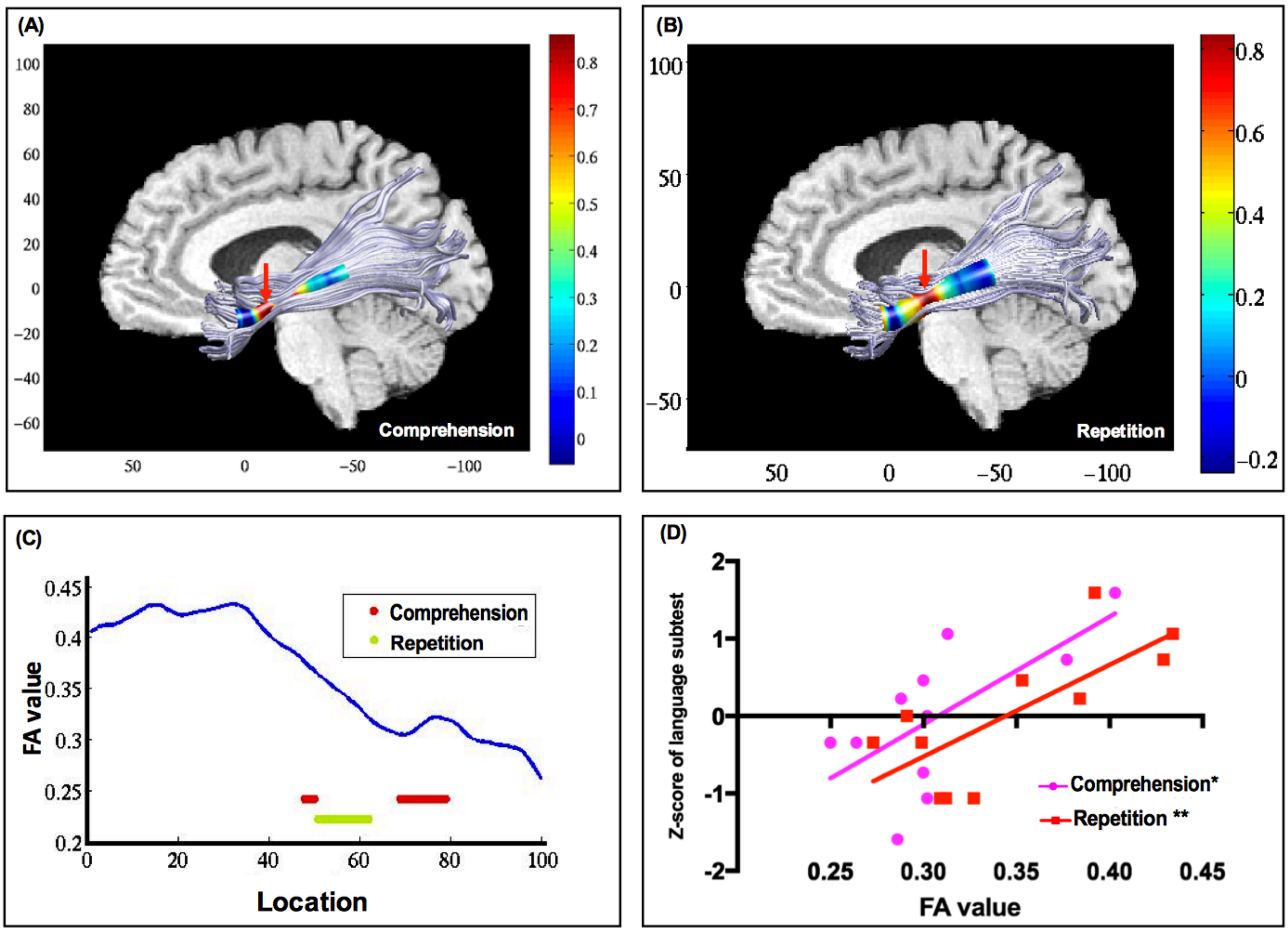

(A)-(B) Colors correspond to the magnitude of correlations along the 100 equidistant points, and red arrows points out the location of the maximal correlation with comprehension and repetition. **(C)** The sections of significant correlations along the 100 equidistant points of the left ILF (all corrected $P < 0.05$), shown as the red bars (comprehension) and yellow bars (repetition). **(D)** Scatter plots with the regression curves for the point of maximal correlation, presenting the linear relationships between FA values (x-axis)

and Z-scores of language subtests on the y-axis (*P<0.05; ** P<0.01). FA, fractional anisotropy; ILF, inferior longitudinal fascicle.

Supplementary Figure 2 Point-wise correlations along the trajectory of the left UF between FA value and language subcomponents.

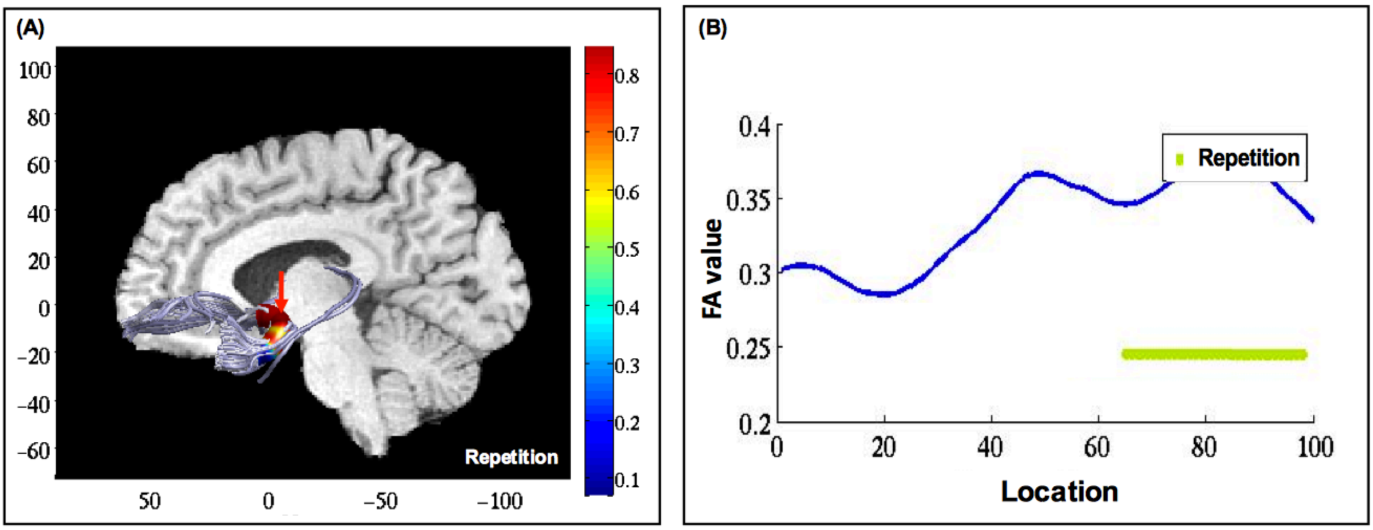

(A) Colors correspond to the magnitude of correlations along the 100 equidistant points, and red arrows points out the location of the maximal correlation with repetition. **(B)** The sections of significant correlations along the 100 equidistant points of the left UF (all corrected P<0.05), shown as the yellow bars (repetition).
